# Targeting B-cell malignancies with the beta-emitting anti-CD37 radioimmunoconjugate ^177^Lu-NNV003

**DOI:** 10.1007/s00259-019-04417-1

**Published:** 2019-07-15

**Authors:** Astri Fjelde Maaland, Helen Heyerdahl, Adam O’Shea, Bergthora Eiriksdottir, Véronique Pascal, Jan Terje Andersen, Arne Kolstad, Jostein Dahle

**Affiliations:** 1 Nordic Nanovector ASA, Kjelsåsveien 168 B, 0884 Oslo, Norway; 2 Institute of Clinical Medicine, University of Oslo, Oslo, Norway; 3 ArcticLAS ehf, Krókhálsi 5d, 110 Reykjavík, Iceland; 4 Centre for Immune Regulation, Department of Immunology, Oslo University Hospital, Rikshospitalet, Oslo, Norway; 5 Department of Pharmacology, Institute of Clinical Medicine, University of Oslo and Oslo University Hospital, Oslo, Norway; 6 Department of Oncology, Oslo University Hospital, Radiumhospitalet, Oslo, Norway; 7 Jebsen Center for Cancer Immunotherapy, University of Oslo, Oslo, Norway

**Keywords:** Radioimmunotherapy, Radioimmunoconjugate, Lutetium-177, Non-Hodgkin’s lymphoma, Chronic lymphocytic Leukaemia, CD37, Diffuse large B cell lymphoma

## Abstract

**Purpose:**

The aim of this study was to explore the β-emitting lutetium-177 labelled anti-CD37 antibody NNV003 (^177^Lu-NNV003, Humalutin®) for the treatment of non-Hodgkin’s lymphoma in *in vitro* studies and in animal models.

**Methods:**

Cytotoxicity of ^177^Lu-NNV003 was measured in REC-1 (mantle cell lymphoma) and DOHH-2 (diffuse large B cell lymphoma) cell lines. Biodistribution was studied in mice bearing subcutaneous DOHH-2 or MEC-2 (chronic lymphocytic leukaemia) xenografts. The therapeutic effect of a single injection of ^177^Lu-NNV003 was measured in mice intravenously or subcutaneously injected with REC-1 cells. Haematological and histopathological assessments were used to evaluate the toxic effect of ^177^Lu-NNV003. The immunotherapeutic effect of NNV003 was assessed by measuring binding to Fcγ receptors, activation of ADCC and ADCP. NNV003’s immunogenicity potential was assessed using in silico immunogenicity prediction tools.

**Results:**

^177^Lu-NNV003 showed an activity dependent antiproliferative effect in all cell lines. Maximum tumour uptake in vivo was 45% of injected activity/g in MEC-2 tumours and 15% injected activity/g in DOHH-2 tumours. In mice injected intravenously with REC-1 cells, ^177^Lu-NNV003 (50–100 MBq/kg) improved survival compared to control groups (*p* < 0.02). In mice with subcutaneous REC-1 xenografts, 500 MBq/kg ^177^Lu-NNV003 extended survival compared to the control treatments (*p* < 0.005). Transient haematological toxicity was observed in all mice treated with radioactivity. NNV003 induced ADCC and ADCP and was predicted to have a lower immunogenicity potential than its murine counterpart.

**Conclusion:**

^177^Lu-NNV003 had a significant anti-tumour effect and a favourable toxicity profile. These results warrant further clinical testing in patients with CD37-expressing B cell malignancies.

**Electronic supplementary material:**

The online version of this article (10.1007/s00259-019-04417-1) contains supplementary material, which is available to authorized users.

## Introduction

Non-Hodgkin’s lymphoma (NHL) accounts for 2.7% of all cancers and 2.4% of all cancer deaths worldwide [1]. Among NHL, diffuse large B cell lymphoma (DLBCL) is the most common subtype, whereas mantle cell lymphoma (MCL) is relatively rare [2]. Chronic lymphocytic leukaemia (CLL), another B cell malignancy, is the most common leukaemia. Chemotherapy is still the cornerstone in the treatment of these diseases, often accompanied by monoclonal antibodies against the CD20 antigen [3–5]. However, targeted therapies like Bruton’s tyrosine kinase (BTK) inhibitors and BCL-2 inhibitors are emerging as novel treatment options, particularly in MCL and CLL [3, 4, 6].

Survival rates for these haematological diseases have improved over recent decades [7–9]. However, many patients will still relapse and become refractory to chemotherapy, anti-CD20 therapy and even the novel targeted treatments [10–12]. Consequently, there is a medical need for new approaches and other targets to overcome resistance. In this study we evaluate a new compound, a next generation radioimmunoconjugate (RIC), for treatment of B cell malignancies. The RIC, ^177^Lu-NNV003, consists of a chimeric mouse-human anti-CD37 antibody (NNV003), conjugated with p-SCN-Bn-DOTA (DOTA) that chelates the β-emitting radionuclide lutetium-177.

Two β-emitting anti-CD20 radioimmunotherapies were approved by the FDA for NHL treatment in the early 2000’s: ^90^Y-ibritumomab tiuxetan (Zevalin®) and ^131^I-tositumomab (Bexxar®). Despite their strong anti-tumour activity these radioimmunoconjugates have not been implemented in clinical practice, as testified by the withdrawal of I^131^-tositumomab in 2014 from the market for business reasons [13].

CD37 is a highly glycosylated transmembrane protein selectively expressed by normal B cells, as well by many B cell malignancies [14]. CD37 has been used as target for naked antibodies and two antibody drug conjugates [15–19]. Furthermore, the next generation RIC (^177^Lu-lilotomab satetraxetan, Betalutin®), which is the murine version of ^177^Lu-NNV003, is currently in clinical trials for relapsed/refractory lymphomas (NCT01796171, NCT02658968) [20, 21] and, based on preclinical data [22], in combination with rituximab for previously treated follicular lymphoma (NCT03806179). In the present study, we have explored the efficacy of unlabelled and lutetium-177 labelled NNV003 chimeric antibody in CLL, MCL and DLBCL models.

## Materials and methods

### Labelling and quality control of antibodies with ^177^Lu

NNV003 (IgG1, mouse variable regions, κ, and human constant region, κ) and cetuximab (Merck KGaA, mouse-human chimeric IgG1, κ) were conjugated with p-SCN-Bn-DOTA (Macrocyclics, Texas, USA) and labelled with ^177^Lu as previously described (see Online Resource for details) [20].

### Cell lines

MEC-2 (CLL, DSMZ GmbH, Braunschweig, Germany) was cultured in IMDM medium; REC-1 (MCL, ATCC, USA) and DOHH-2 (DLBCL, DSMZ GmbH, Braunschweig, Germany) were cultured in RPMI medium. The media were supplemented with 10% heat inactivated fetal bovine serum and 1% penicillin-streptomycin (Thermo Fisher Scientific, Massachusetts, USA).

### Binding of NNV003 and lilotomab to effector molecules

The binding properties of NNV003 and lilotomab to human and mouse Fcγ receptors (hFcγRs and mFcγRs) and to the neonatal Fc receptor (FcRn) were evaluated by ELISA as previously described [23].

### ADCP and ADCC assays

Antibody-dependent cellular phagocytosis (ADCP) and antibody-dependent cellular cytotoxicity (ADCC) induction in MEC-2, REC-1 and DOHH-2 cell lines were measured using an ADCP- and ADCC Reporter Bioassay kit (Promega, USA) according to the manufacturer’s instructions (see Online Resource for details).

### Cell cytotoxicity of ^177^Lu-NNV003

Cell proliferation after treatment with unlabelled or ^177^Lu labelled NNV003, or unspecific isotype antibody (^177^Lu-cetuximab) was measured in REC-1 and DOHH-2 using CyQUANT™ NF Cell Proliferation Assay Kit (Thermo Fisher Scientific, USA) (see Online Resource for details).

### Animals

Animal studies were approved by the National Animal Research Authorities and carried out according to the European Convention for the Protection of Vertebrates Used for Scientific Purposes regulations. All mice were housed under pathogen free condition and had ad libitum access to food and water. In all animal models health and body weight conditions were monitored using a score system to track symptoms severity. Animals were euthanized when a humane end-point was reached.

### Biodistribution of ^177^Lu-NNV003

Biodistribution of ^177^Lu-NNV003 was performed in female NSG mice (NOD.Cg-*Prkdc*^*scid*^*Il2rg*^*tm1Wjl*^/SzJ; The Jackson Laboratory, USA, 6–7 weeks old, average weight 20.6 g) with MEC-2 subcutaneous (s.c.) xenografts, and in female and male CB17 SCID mice (CB-17/Icr-*Prkdc*^*scid/scid*^/Rj, Janvier Labs, France, 10 weeks old, average weight 21.7 g) with DOHH-2 s.c. xenografts. Mice were injected with 10 × 10^6^ MEC-2 cells or 5 × 10^6^ DOHH-2 cells in each flank. Mice were injected with 1 MBq (MEC-2) or 1.2 MBq (DOHH-2) of ^177^Lu-NNV003 in 100 µl formulation buffer when tumours were of suitable size. The day prior to injection of RIC, 200 μg mouse IgG2a-κ (M7769-5MG, Sigma-Aldrich, USA) was administered intraperitoneally (i.p.). Mice were necropsied at different time points after RIC injection. Radioactivity was measured by gamma counters (Wizard 3470, PerkinElmer, USA or Cobra II auto-gamma detector, Packard Instrument Company, USA). Samples from the injected solution of ^177^Lu-NNV003 were used as references in the measurement procedures.

### Radiation dosimetry of ^177^Lu-NNV003

The biodistribution data from the DOHH-2 s.c. model was used to calculate the absorbed radiation doses from ^177^Lu-NNV003 in different organs as previously described [24] (see Online Resource for details).

### Therapeutic effect of NNV003 and ^177^Lu-NNV003

To assess the anti-tumour effect of the unlabelled NNV003 compared to lilotomab in vivo, female CB17 SCID mice (C.B-*Igh-1*^*b*^/IcrTac-*Prkdc*^*scid*^, Taconic, Denmark, 8 weeks old, average weight 18.5 g) were injected intravenously (i.v.) with 10 × 10^6^ REC-1 cells one day prior to treatment; 100 μg antibodies were injected i.v. twice a week for four weeks (*n* = 10 mice per group). Humane end-points were > 10% weight loss/gain over a period of one week, palpable tumour > 12 mm, or signs of substantial discomfort. The remaining mice were then euthanized and necropsied 166 days after cell injection.

Anti-tumour effect of a single injection ^177^Lu-NNV003 was investigated in female CB17 SCID mice (CB-17/Icr-*Prkdc*^*scid/scid*^/Rj, Janvier Labs, France, 9–10 weeks old, average weight 20 g), who were injected i.v. with 20 × 10^6^ REC-1 cells two days prior to therapy injections. Six groups (*n* = 8) were treated with 0.9% NaCl, 0.167 mg/kg unlabelled isotype control (cetuximab), 100 MBq/kg ^177^Lu labelled cetuximab, 0.167 mg/kg NNV003, 50 MBq/kg or 100 MBq/kg ^177^Lu-NNV003. Mouse IgG2a-κ was injected i.p. the day before therapy injection. Humane end-points were hind leg paralysis, weight loss > 15%, palpable tumour > 15 mm or any signs of severe sickness or discomfort. The remaining mice were then euthanized and necropsied 169 days after cell injection.

To compare the anti-tumour effect of ^177^Lu-NNV003 and NNV003, therapies were tested in a s.c. model where 10 × 10^6^ REC-1 cells were injected s.c. in each flank of female nude mice (Hsd:Athymic Nude-*Foxn1*^*nu*^, Envigo, France, 8 weeks old, average weight 22.0 g). To ensure tumour growth, 80 μl Anti-asialo (diluted in 2 ml, Wako Chemicals, USA) was injected i.p. at day −1, 4 and 8 from cell inoculation. When the average tumour size was 173 mm^3^ 13 days after cell inoculation, mice were treated with 0.9% NaCl, 10 mg/kg unlabelled isotype control (cetuximab), 500 MBq/kg ^177^Lu labelled isotype control (^177^Lu-cetuximab), 1.6 or 10 mg/kg NNV003, or 500 MBq/kg ^177^Lu-NNV003 (*n* = 10). Mouse IgG2a-κ was injected i.p. the day before therapy injection. Humane end-point was reached when total tumour volume was 2500 mm^3^ (tumour burden within 10% of body weight) or ulcer on the tumour surface.

GraphPad Prism 7.00 (GraphPad Software, La Jolla California, USA) was used to create Kaplan-Meier survival curves and to perform comparisons of the groups using Log-rank test and Holm-Sidak method with α = 0.05.

### Toxicity of ^177^Lu-NNV003

Female Rag2 deficient mice (129S6/SvEvTac-*Rag2*^*tm1Fwa*^, Taconic, USA, 9–13 weeks old, average weight 20.5 g) were injected i.v. with 10 × 10^6^ DOHH-2 cells three days prior to therapy injections. Mice (*n* = 9 or 10) were treated with 200, 300 or 400 MBq/kg ^177^Lu-NNV003, 2 or 30 mg/kg unlabelled NNV003, 0.9% NaCl, 2 mg/kg unlabelled or 300 MBq/kg ^177^Lu labelled cetuximab. Mouse IgG2a-κ was injected i.p. the day before therapy injection. Blood was sampled in three to five mice in each group 2.5 weeks before therapy injection and 1.5, 3, 4.5, and 6 weeks after. Red blood cell, white blood cell and platelet concentrations were measured using a haematology analyser (MS4 analyser, Melet Schloeing Laboratories, France). Serum was collected when the animals were euthanized and alanine aminotransferase (ALT), alkaline phosphatase (ALP), urea and creatinine were measured on a VetScan VS2 (Abaxis, USA). One-way ANOVA was performed in GraphPad Prism 7.00 (GraphPad Software, La Jolla California, USA) to test for significant differences. Histopathological examinations were performed on selected organs.

## Results

### Characterisation of NNV003

**Fig. 1:**
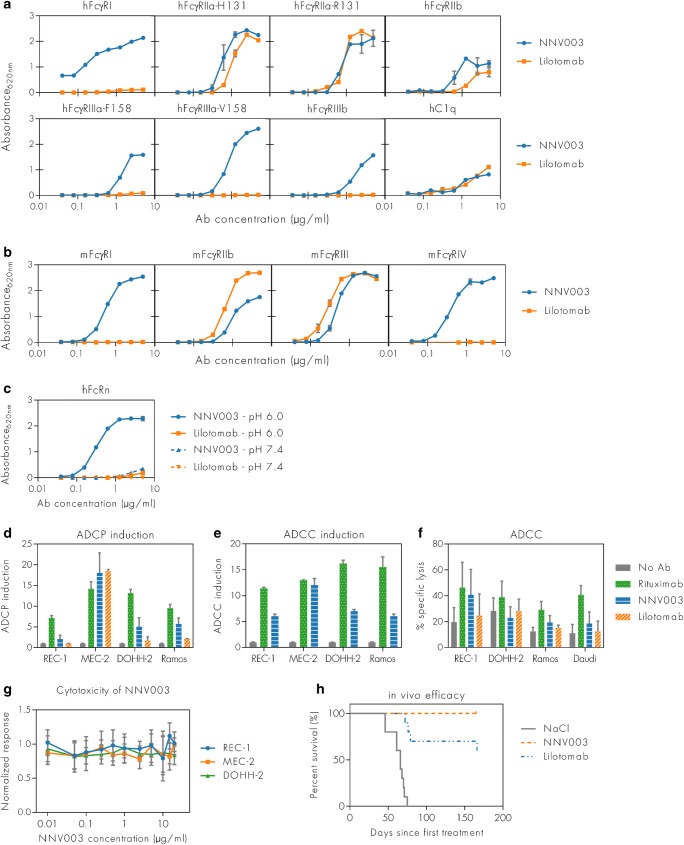
Comparison of NNV003 and lilotomab with respect to effector molecules binding, ADCP, ADCC, cytotoxicity and therapeutic effect in vivo. Binding of NNV003 and lilotomab to (**a**) hFcγRs, (**b**) mFcγRs and (**c**) human FcRn at neutral and acidic pH. (**d**) ADCP and (**e**) ADCC induction of NNV003, lilotomab and rituximab in different target cells. (**f**) Specific lysis of four target cell lines by ADCC of NNV003, lilotomab and rituximab. (**g**) Cytotoxicity of NNV003 and (**h**) in vivo efficacy of 100 μg NNV003 and lilotomab, injected twice a week for 4 weeks, in CB17 SCID mice i.v. injected with REC-1 cells

The binding properties of NNV003 and its mouse counterpart lilotomab to the classical human and murine forms of the FcγRs are shown in Figs. 1a and b. Generally, NNV003 bound more strongly than lilotomab to most of the activating hFcγRs, while lilotomab did not bind to hFcγRI, hFcγRIIIa and hFcγRIIIb. Both antibodies bound weakly to human C1q. Lilotomab bound more strongly than NNV003 to mFcγRIIb and mFcγRIII. However, NNV003, but not lilotomab, bound to mFcγRI and mFcγRIV. Only NNV003 bound pH dependently to human FcRn while lilotomab did not bind (Fig. 1c), which is in line with the fact that mouse IgG subclasses do not bind significantly to the human receptor, and as such NNV003 and not lilotomab is expected to be rescued from degradation via FcRn in vivo [25, 26], indicating that the biological half-life of NNV003 will be longer than for lilotomab.

Both ADCC and ADCP induction was highly dependent on target cell line (Fig. 1d and e). The level of induction by NNV003 was comparable to that of rituximab (positive control) in MEC-2 cells, but generally lower in other target cells. Cell lysis by ADCC by both NNV003 and lilotomab was low compared with rituximab and comparable to the no antibody control in most of the target cell lines (Fig. 1f).

No cytotoxic effect of NNV003 alone was observed in vitro (Fig. 1g); however, treatment with 100 μg NNV003 twice a week for four weeks resulted in 100% survival of mice i.v. injected with REC-1 cells with more than 150 days observation time (Fig. 1h).

The predicted immunogenicity potential of NNV003 was lower than for lilotomab (Table 1, Fig. S1 in Online Resource).

**Table 1:** Immunogenicity risk scores for NNV003 and lilotomab

Antibody	Immunogenicity risk score		
	Light chain	Heavy chain	Total
Lilotomab	15.83	20.54	36.37
NNV003	15.81	7.34	22.52

### Anti-proliferative effect, binding and internalisation of ^177^Lu-NNV003 in vitro

**Fig. 2:**
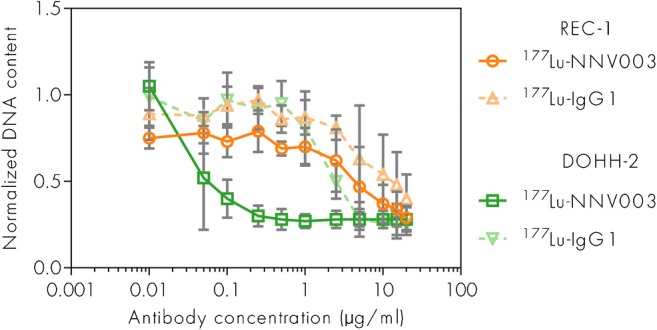
Anti-tumour effect of ^177^Lu-NNV003 in vitro. Proliferation of REC-1 and DOHH-2 cells treated with ^177^Lu-NNV003 or ^177^Lu-IgG1 (*n* = 3, error bars = SD) for 20 h, then washed and measured after 6 days. Normalized against untreated control cells

^177^Lu-NNV003 showed an anti-proliferative effect against the cell lines tested (Fig. 2). However, only in DOHH-2 cells did ^177^Lu-NNV003 significantly inhibit proliferation more than the ^177^Lu-IgG1 control, indicating that the radiation from the unbound activity in the serum was important for the anti-proliferative effect. Binding studies with ^177^Lu-NNV003 showed that the antibody bound to REC-1, MEC-2 and DOHH-2 with an affinity of approximately 1 nM and that the cell lines expressed 60 000 (REC-1), 80 300 (MEC-2) and 104 000 CD37 antigens per cell (Table S1 in Online Resource). After 18 h of incubation, REC-1 had internalised 37% of the bound ^177^Lu-NNV003, MEC-2 had internalised 14% and DOHH-2 6% (Fig. S2 in Online Resource).

### Biodistribution and radiation dosimetry of ^177^Lu-NNV003

**Fig. 3:**
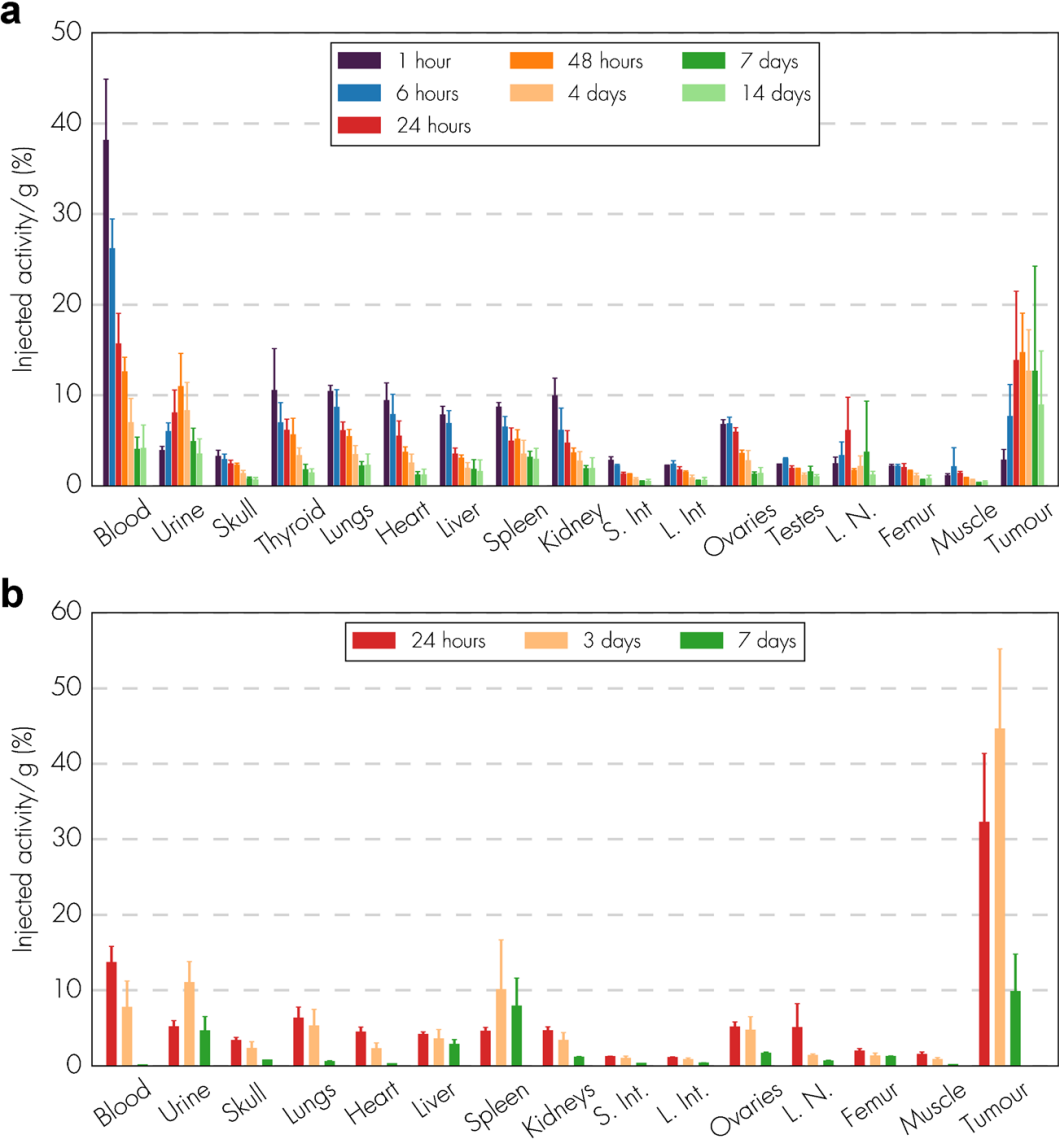
Biodistribution of ^177^Lu-NNV003. (**a**) CB17 SCID mice with DOHH-2 s.c. xenografts (*n* = 6 mice per time point or n = 3 (ovary and testis), error bars = SD) were injected i.v. with 1.2 MBq ^177^Lu-NNV003 and (**b**) NSG mice with MEC-2 s.c. xenografts (n = 3 mice per time point, error bars = SD) were injected i.v. with 1 MBq ^177^Lu-NNV003. Tissues were harvested and measured at different time points. S. Int = small intestines, L. int = large intestines, L.N. = Lymph nodes

**Fig. 4:**
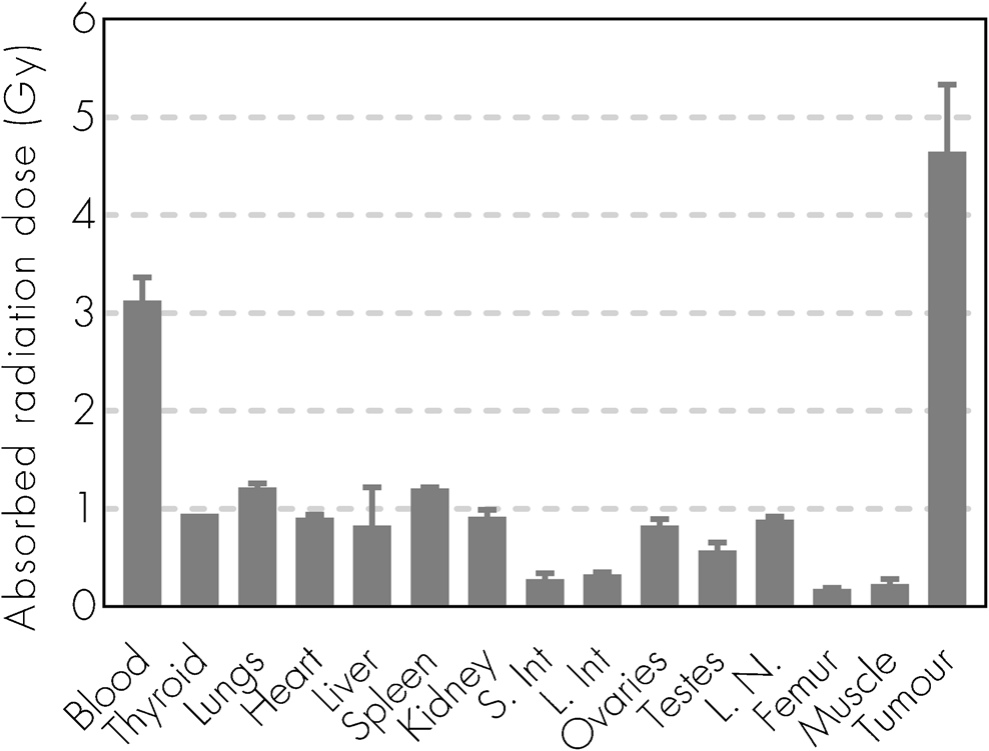
Dosimetry of ^177^Lu-NNV003. Absorbed radiation dose (Gy) to organs and tumour for CB17 SCID mice with DOHH-2 s.c. xenografts injected with ^177^Lu-NNV003 (n = 6 mice or n = 3 (ovary and testis) per time point, error bars = SD). The data was normalized to an injected activity of 100 MBq/kg. S. Int = small intestines, L. int = large intestines, L.N. = Lymph nodes

The biodistribution of ^177^Lu-NNV003 was performed in NSG mice with s.c. MEC-2 xenografts and in CB17 SCID mice with s.c. DOHH-2 xenografts (Fig. 3). The day prior to RIC injection, 200 μg mouse IgG2a-κ was administered i.p. to inhibit the binding of ^177^Lu-NNV003 to murine Fc receptors and thus to prevent clearance of antibodies in the spleen and liver. Mouse IgG2a binds with a similar affinity to murine Fc receptors as human IgG1 [27–29]. ^177^Lu-NNV003 reached a maximum uptake of 45% of injected activity/g in MEC-2 tumours after three days and 15% in DOHH-2 tumours after two days. The retention of ^177^Lu-NNV003 in tumour was more stable for DOHH-2 tumours than for MEC-2; the injected activity/g was approximately 12% for DOHH-2 and 10% for MEC-2 7 days after injection. After initial distribution to normal organs, no redistribution of the radionuclide was observed. The absorbed radiation dose after injection of 100 MBq/kg ^177^Lu-NNV003 in DOHH-2 tumours was 4.6 Gy and lower than 1.2 Gy in all of the normal tissues, except in blood (Fig. 4).

### Therapeutic effect of ^177^Lu-NNV003

**Fig. 5:**
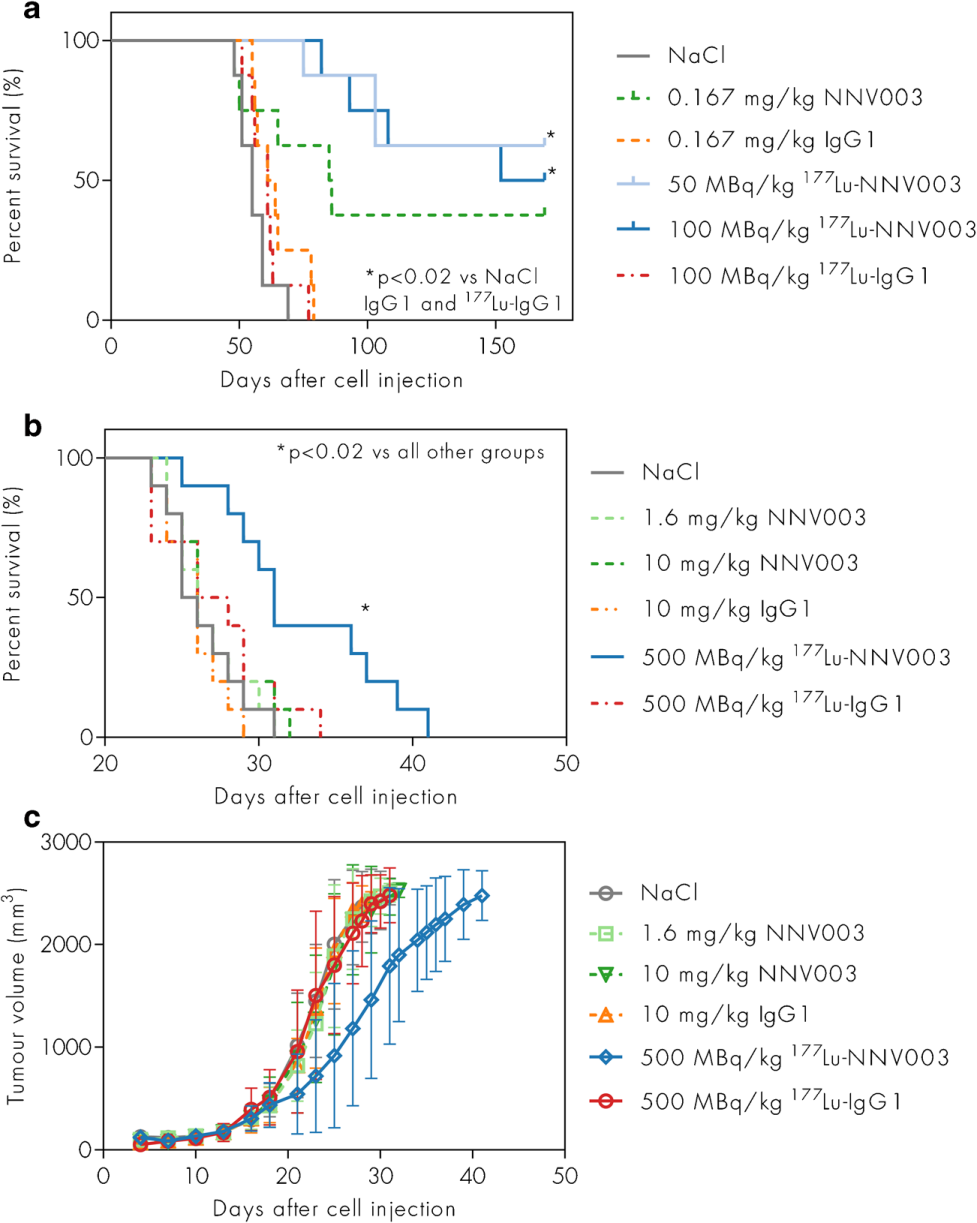
Anti-tumour effect of ^177^Lu-NNV003 in vivo. Survival of (**a**) CB17 SCID mice (*n* = 8 per group) treated with NaCl, unlabelled unspecific IgG1 or NNV003, ^177^Lu-IgG1 or ^177^Lu-NNV003 two days after i.v. injection of REC-1 cells. Mice that survived to the end of the study are censored. Survival (**b**) and tumour growth (**c**) of nude mice (*n* = 10 per group) with s.c. REC-1 xenografts treated with NaCl, unlabelled unspecific IgG1 or NNV003, ^177^Lu-IgG1 or ^177^Lu-NNV003. All mice in (**b**) reached end-point criteria and none are censored

In CB17 SCID mice i.v. injected with REC-1 cells two days prior to therapy, improved survival was observed in animals treated with ^177^Lu-NNV003 (50 and 100 MBq/kg) compared to the IgG1 and the NaCl control groups (*p* < 0.02) (Fig. 5a). NNV003 had a modest anti-tumour effect, but survival was not significantly prolonged compared to the NaCl and IgG1 control groups (*p* > 0.15). At the end of the study, 38% of the mice treated with NNV003 survived, while 63% and 50% in the 50 and 100 MBq/kg ^177^Lu-NNV003 groups survived, respectively. There was no statistically significant difference between the groups treated with NNV003 or ^177^Lu-NNV003 (*p* > 0.55). Histopathological examination of normal organs from surviving mice showed no signs of tumour infiltration (data not shown), while tumour infiltration in the pelvic region, abdomen, ovaries and skull was confirmed by autopsy in the non-responding mice euthanized before the end of the study.

Two additional therapy studies in i.v. models were performed (Fig. S3 in Online Resource). In a MEC-2 i.v. model, only the treatment with ^177^Lu-NNV003 increased survival compared with control groups, but in a DOHH-2 i.v. model, NNV003 and ^177^Lu-NNV003 treatments were equally efficient in extending survival of the mice.

Since the range of β particles is more suited to treat bulky tumours, a therapy study in nude mice with REC-1 s.c. tumours was performed. A dose of 500 MBq/kg ^177^Lu-NNV003 significantly increased survival and slowed down tumour growth, compared to control treatment and to NNV003 treatment (*p* < 0.005), even at high doses of 10 mg/kg of NNV003 (Fig. 5b and c).

### Toxicity of ^177^Lu-NNV003

**Fig. 6:**
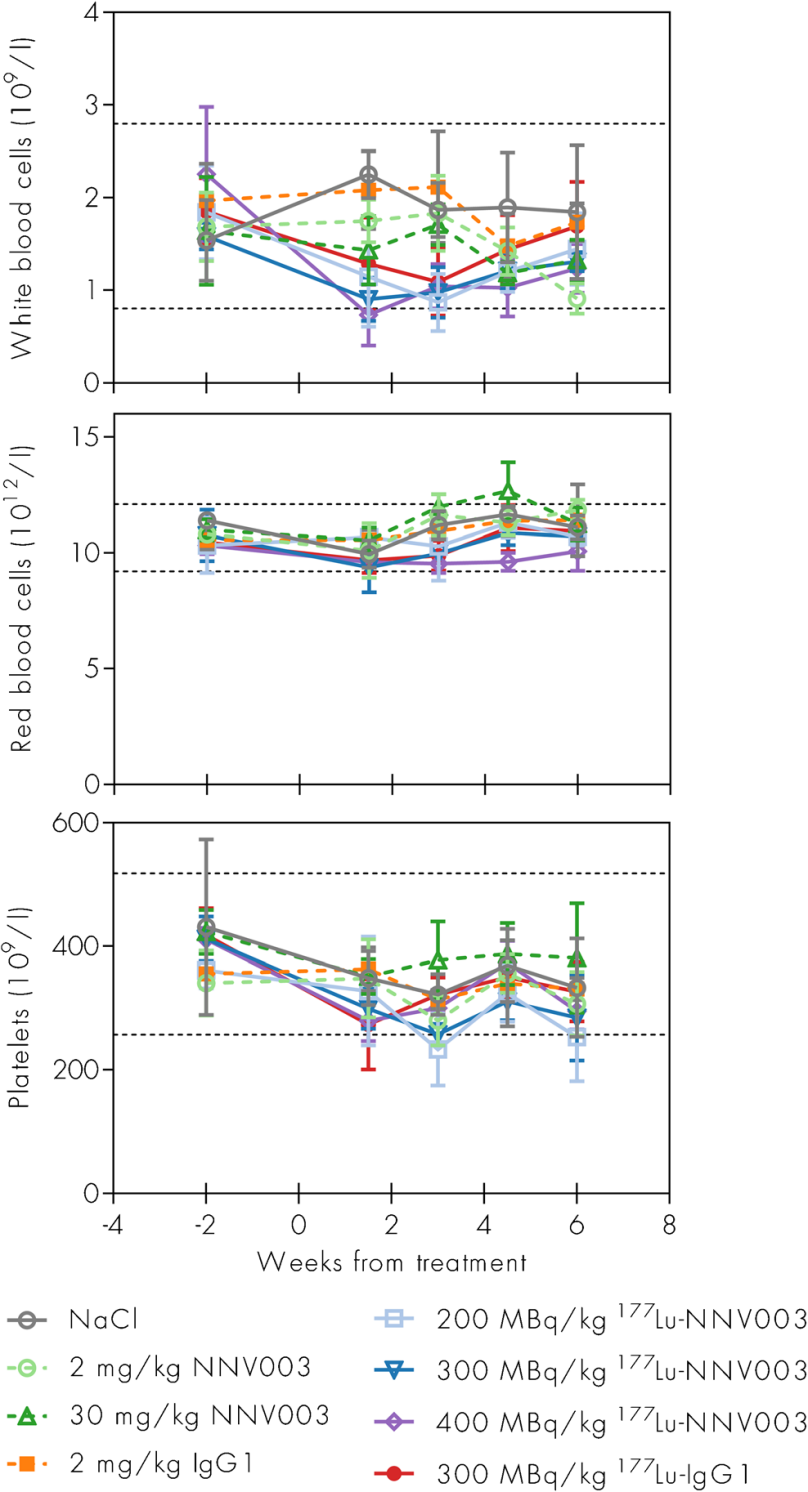
Haematological toxicity of ^177^Lu-NNV003. White blood cells, red blood cells and platelet concentrations in Rag2 mice i.v. injected with DOHH-2 cells three days before treatment with unlabelled unspecific IgG1 or NNV003, NaCl or lutetium-177 labelled unspecific IgG1 or NNV003. Average of 3–5 mice per group, error bars = SD. *Dotted lines* show reference intervals calculated using Reference Value Advisor [43] from haematology measurements from 39 Rag2 mice

Female Rag2 mice were injected i.v. with DOHH-2 cells three days prior to therapy with 200–400 MBq/kg ^177^Lu-NNV003 or control treatments. A modest and temporary decrease in white blood cell count was observed in the groups treated with ^177^Lu-NNV003 or ^177^Lu-IgG1 (Fig. 6), while platelet and red blood cell counts did not significantly change.

**Fig. 7:**
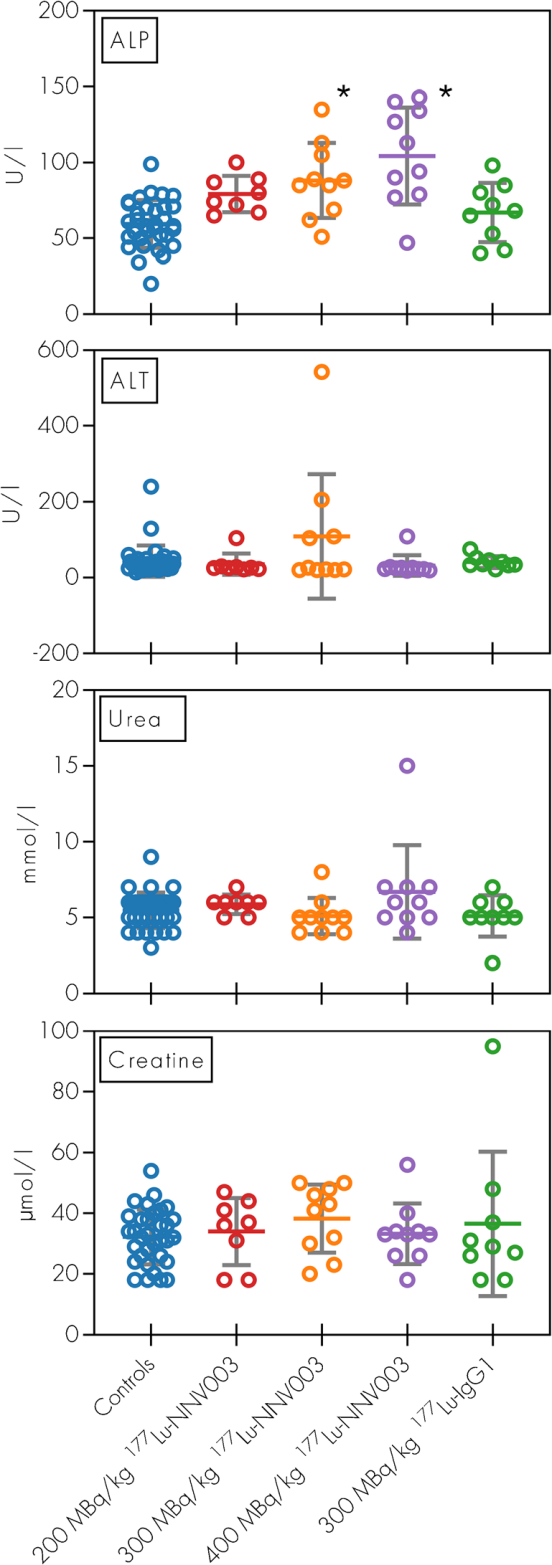
Clinical chemistry in mice treated with ^177^Lu-NNV003. ALT, ALP, urea and creatinine serum concentrations of Rag2 mice i.v. injected with DOHH-2 cells three days before treatment. Data from cold antibody groups and NaCl groups showed no difference and were pooled together (Controls). Serum samples were collected at euthanasia. Individual data plotted as points. Asterisk: *p* < 0.001 compared to controls

Mice treated with 300 and 400 MBq/kg ^177^Lu-NNV003 showed a modest increase in both ALP (significant difference) and ALT (non-significant difference) compared to control mice, indicating slight hepatotoxicity (Fig. 7). Levels of urea and creatinine indicated no signs of renal toxicity. All mice survived until the end of the study without clinical signs of disease, and no histopathological abnormalities were found in any of the potentially affected organs. Atrophy, interstitial cell hyperplasia and a low incidence of cysts were observed in the ovaries in ^177^Lu-NNV003 treated mice (data not shown).

## Discussion

Despite the advances in NHL treatments in recent years, there is still an unmet medical need for patients resistant to chemotherapy, CD20 immunotherapies and kinase inhibitors. The mouse-human IgG1 chimeric anti-CD37 RIC ^177^Lu-NNV003 represents a new treatment option with a different target and mechanism of action. Here we show that ^177^Lu-NNV003 increased survival of mice with xenografts of human CLL, MCL and DLBCL. The chimeric NNV003 antibody targets CD37 positive cells both in vitro and in vivo.

The mouse monoclonal antibody lilotomab has been shown to bind CD37 on normal B cells and B cell malignancies with a high level of specificity [20]. NNV003 is a mouse-human chimeric IgG1 version of lilotomab and binds to the same epitope on CD37. The higher anti-tumour effect of NNV003 than of lilotomab in the mouse models is likely due to stronger binding of NNV003 to the mFcγRs. Stronger binding of mouse-human chimeric IgG1 antibodies than of mouse antibodies to the mFcγRs is consistent with other studies [27, 28].

The anti-proliferative effect of ^177^Lu-NNV003 on the cell lines in vitro was most pronounced for DOHH-2, which may be due to the higher CD37 expression on DOHH-2 compared to REC-1. In REC-1 the cytotoxic effect of ^177^Lu-NNV003 differed only slightly from that of lutetium-177 labelled unspecific antibody. This unspecific effect was probably due to cross-irradiation from unbound conjugate during the incubation period before washing the cells [30]. In the in vivo studies, however, ^177^Lu-IgG1 did not improve survival.

A high uptake of ^177^Lu-NNV003 in CD37-expressing tumours compared to normal organs and blood was observed. The higher uptake in MEC-2 tumours than in DOHH-2 tumours, even though the CD37 expression was similar, might be explained by higher degree of ^177^Lu-NNV003 internalisation in MEC-2 cells (Online Resource, Table S1). Biodistribution and dosimetry were not performed for REC-1, but it is reasonable to expect similar results for this animal model.

In an i.v. REC-1 xenograft model, CB17 SCID mice treated with ^177^Lu-NNV003 showed prolonged survival compared to controls. Some efficacy was also observed with NNV003, probably due to an immunotherapeutic effect of the antibody. In another i.v. model using DOHH-2 cells, the anti-tumour effect of NNV003 was equal to the effect of ^177^Lu-NNV003 (Online Resource, Fig. S3b). However, in a s.c. model mimicking a bulkier disease, there was no effect of NNV003 alone, even at a clinically relevant dose of 10 mg/kg. In this model, the immunotherapeutic effect was not potent enough, while ^177^Lu-NNV003 delayed tumour growth and prolonged survival significantly. All animal models utilized mouse strains with functional NK-cells and macrophages, capable of ADCC and ADCP activity [31, 32].

We observed a modest and temporary reduction in white blood cell counts after ^177^Lu-NNV003 treatment. Additionally, while some biochemical indication of hepatotoxicity was seen, no abnormal findings of the liver were observed by histopathological examination of organs. In the present study, treatment related changes were mainly seen in the ovaries, consistent with pre-clinical studies with ^177^Lu-lilotomab satetraxetan [33]. No treatment related changes were identified in the bone marrow, possibly attributed to bone marrow regeneration due to the long study duration. In a phase 1/2a clinical trial of ^177^Lu-lilotomab satetraxetan for treatment of patients with relapsed indolent NHL, reversible thrombocytopenia and neutropenia were the most common adverse events observed [21].

The favourable efficacy and toxicity profile of ^177^Lu-NNV003 suggests a beneficial therapeutic window. Moreover, this compound binds to a different antigen than CD20, which is the most common target in the treatment of B cell malignancies. Accordingly, most NHL patients with recurrent disease will become refractory to treatments targeting CD20 [12]. By pursuing a different target, like CD37, ^177^Lu-NNV003 may overcome this resistance and become a valid option for patients with recurrent disease. Other novel treatments like BTK inhibitors and BCL2-inhibitors have offered effective treatment options, particularly for patients with CLL and MCL [34–40]. However, most patients will eventually relapse again and ^177^Lu-NNV003 might then represent a tolerable and effective treatment option.

The absorbed doses to tumours (4.6 Gy in DOHH-2) and normal tissues are consistent with that of ^177^Lu labelled lilotomab in in vivo models [24]. The range of the tumour doses (0.75–7.94 Gy) reported clinically with ^177^Lu-lilotomab satetraxetan, resulted in an overall response rate of 61% and 28% complete response [21, 41], indicating that the absorbed radiation doses were high enough to achieve a clinical response. This is in line with a previous report showing that 4 Gy of external beam radiotherapy is very effective for local control in patients with indolent NHL [42].

In conclusion, the next generation chimeric anti-CD37 RIC ^177^Lu-NNV003 was shown to improve survival of mice with CLL, MCL and DLBCL xenografts and to have a favourable toxicity profile. These encouraging results warrant further investigation of ^177^Lu-NNV003 in clinical trials to determine whether the additional immunotherapeutic component, longer biological half-life and potential reduced risk of immunogenicity offers any clinical advantage to patients with relapsed B cell malignancies.

## Electronic supplementary material

ESM 1 (PDF 245 kb)
