# Supplementary material for: Targeting B-cell malignancies with the beta-emitting anti-CD37 radioimmunoconjugate 177Lu-NNV003
Source: Eur J Nucl Med Mol Imaging. 2019 Jul 15;46(11):2311–21. doi: 10.1007/s00259-019-04417-1 (PMC6717602; doi:10.1007/s00259-019-04417-1)
Supplement: Supplementary file 1 — (PDF 245 kb) [file 259_2019_4417_MOESM1_ESM.pdf]

Supplementary Material for
Targeting B-cell malignancies with the beta-emitting anti-CD37 radioimmunoconjugate ^{177}Lu-NNV003

Contents

Detailed Materials and Methods 2

Labelling and Quality Control of antibodies with ^{177}Lu 2

ADCP and ADCC assays 2

Cell cytotoxicity of ^{177}Lu-NNV003 3

Radiation dosimetry of ^{177}Lu-NNV003 3

Additional data 4

In silico Immunogenicity prediction 4

Binding of ^{177}Lu-NNV003 to CD37 positive cell lines 6

Internalisation of ^{177}Lu-NNV003 6

Additional ^{177}Lu-NNV003 therapy studies 7

References 9

Detailed Materials and Methods

Labelling and Quality Control of antibodies with ^{177}Lu

NNV003 and cetuximab (Merck KGaA, used as isotype control), both chimeric IgG1 antibodies, were first conjugated with p-SCN-Bn-DOTA (Macrocyclics, Texas, USA) dissolved in 5 mM HCl. p-SCN-Bn-DOTA was added in carbonate buffer pH 8.8, at a molar ratio of 10:1. The solution was incubated at 37°C while shaking for 2 hours, before it was stopped by addition of 50 µl of 0.2 M glycine solution (Merck Millipore, Massachusetts, USA) per mg of antibody. A buffer exchange with 50 ml 0.9% NaCl was performed by centrifugation (Centrifuge 5804R, Eppendorf, Germany) in Vivaspin 15R centrifuge tubes (Sartorius, Goettingen, Germany).

The pH of DOTA-NNV003 and DOTA-cetuximab were adjusted to 5.4 using 0.25 M ammonium acetate buffer before adding ^{177}Lu in 10 mM HCl (ITG, Garching, Germany) to obtain specific activities of approximately 200 MBq/mg (biodistribution studies) or 600 MBq/mg conjugate (cytotoxicity assay), 420 MBq/mg (REC-1 intravenous therapy study) and 350 MBq/mg (REC-1 subcutaneous therapy study). The samples were incubated for 30 min at 37°C and stopped by addition of formulation buffer composed of PBS containing 7.5 %v/v recombinant albumin (Novozymed Biopharma, Denmark), 200 mM sodium ascorbate (Apotekproduksjon AS, Norway) and 10 mM DTPA (Heyl Pharm.-Chem. Fabrik, Germany) adjusted to pH 7.5.

Instant thin layer chromatography (Tec-Control ITLC strips, Biodex Medical, New York, USA) was used to measure the radiochemical purity (RCP) of the conjugates. If the RCP was below 95%, the conjugate was purified using Sephadex G-25 PD-10 columns (GE Healthcare Life Sciences, Chicago, USA) and RCP >95% was verified. The immunoreactivity was measured using a modified Lindmo model [1] with one cell concentration of 75 x 10^6 cells/ml. The immunoreactivities of ^{177}Lu-NNV003 used in the experiments were between 70% and 88%.

ADCP and ADCC assays

A FcγRIIa-H ADCP- and a FcγRIIIa ADCC Reporter Bioassay kit (Promega, USA) were used for measuring ADCP and ADCC induction in MEC-2, REC-1 and DOHH-2. 2.5 µg/ml NNV003, lilotomab or rituximab (Roche, Switzerland, used as positive control) was added to the target cells (1 x 10^6 cells/ml), using three replicates per antibody per cell line. Effector cells were added at a 2:1 effector:target cell ratio and the cells incubated for 21 hours. After bioluminescence measurements, the background signal was subtracted from each well, and the wells were normalized against the control containing only target and effector cells.

Cell lysis by ADCC was measured by chromium-51 release assay. Target cells, REC-1, DOHH-2, Ramos (Burkitt's lymphoma) and Daudi (Burkitt's lymphoma) were labelled with ^{51}Cr and incubated with NNV003 or lilotomab (20 µg/ml) or rituximab as positive control (10 µg/ml) for 10 min. IL-2 stimulated human PBMC was added at a 10:1 effector:target cell ratio. After 4 hours of incubation the cells were washed, and the cytotoxic effect was measured by Cr^{51} release into the supernatant by a gamma counter (Cobra II, Packard, Perkin Elmer, USA). Controls: target cells were cultivated in medium alone (spontaneous lysis) and in medium containing 1% Triton X-100 (maximal lysis). Specific lysis was calculated by subtracting the spontaneous release from the experimental release and dividing by the maximum release minus the spontaneous release.

Cell cytotoxicity of ^{177}Lu-NNV003

Cell proliferation after treatment with unlabelled or ^{177}Lu labelled NNV003, or unspecific isotype antibody (^{177}Lu-cetuximab) was measured in REC-1 and DOHH-2. Cells were seeded in deep well plates at a density of 1.9–2.7 x 10^6 cells/ml. Antibodies were added to the cells at final concentrations of 10 ng/ml to 20 µg/ml, using two replicates per cell line per treatment. The cells were incubated while shaking for 20 hours and then washed three times in PBS containing 0.5% bovine serum albumin (BSA). After resuspension in cell culture medium to 0.4 x 10^6 cells/ml, the cells were re-seeded in 96-microwell plates for a further six days of growth. Cells treated with unlabelled NNV003 were not washed, only diluted to 0.4 x 10^6 cells/ml. CyQUANT™ NF Cell Proliferation Assay Kit (Thermo Fisher Scientific, USA) was used to measure cell proliferation. The experiments were performed on 2 or 3 separate occasions. The cell lines had a doubling time of approximately 2 days.

Radiation dosimetry of ^{177}Lu-NNV003

The biodistribution data from the DOHH-2 s.c. model was used to calculate the absorbed radiation doses from ^{177}Lu-NNV003 in different organs. The area under the activity versus time curves (AUCs) were normalized to an injection of 100 MBq/kg, the activity used in the REC-1 i.v. therapy study. AUCs, calculated by the trapezoidal rule, were multiplied by the mean energy of the β-particles, Auger- and conversion electrons of 0.147 MeV [2]. At t=0 100% of the injected activity was estimated to be in the blood. The absorbed radiation doses were adjusted for self- and cross-radiation by multiplying by factors developed by Miller et al., 2005 [3]. The self-radiation of blood and brain was set to 1. For lymph nodes, factors developed in [3] for a tumour of 25 mg was used. The factor used for self-radiation for the tumours was 0.95, according to the average tumour mass in the study (0.3 g).

Additional data

In silico Immunogenicity prediction

In silico Major Histocompatibility Complex (MHC) class II-peptide binding predictive analysis of NNV003 and lilotomab was performed by EIR Science A/S (Copenhagen, Denmark) using the NetMHCIIpan 3.1 algorithm [4]. Only peptides predicted with a rank score of < 10 were considered as potential MHC class II binders. Immunogenicity risk scores (IRS) were calculated based on MHC class II binding profiles of protein derived 15-mer peptides to a "world average" population of MHC class II alleles consisting of more than 300 different MHC Class II alleles and 11 geographical defined populations. The IRS reflects the number of MHC class II-binding peptides weighted by the frequency in the investigated population. Self-adjusted IRS were obtained after subtraction of the epitopes also found in known antibody germline sequences and antibody conserved regions.

NNV003 is a mouse-human chimeric IgG1 monoclonal antibody (mAb), containing the variable heavy (V_H) and light (V_L) regions of its murine analogue, lilotomab, a mouse IgG1, κ (mIgG1, κ) mAb, and the constant regions of the heavy (C_H) and light (C_L) chains of human IgG1, κ (hIgG1, κ). In silico MHC class II-binding prediction analysis of peptides derived from NNV003 light and heavy chain sequences suggests that replacing lilotomab (C_H+C_L) with hIgG1 (C_L+C_H) decreases NNV003 immunogenicity potential, by removing the predicted T cell neo-epitopes identified in lilotomab C_H and C_L (see Figure S1, red profile, lilotomab only). It is of interest to note that this decrease of immunogenicity potential is mainly the consequence of T cell neo-epitopes removal in lilotomab C_H, as replacing lilotomab C_L with the hIgG1, κ counterparts generates a new promiscuous overlapping T cell neo-epitope spanning NNV003 V_L-C_L in position 102-116 (see Figure S1, NNV003 blue immunogenicity profile not overlapping lilotomab red immunogenicity profile). Of note, this T cell neo-epitope is also identified in the rituximab light chain sequence overlapping the murine V_L-human C_L region [5].

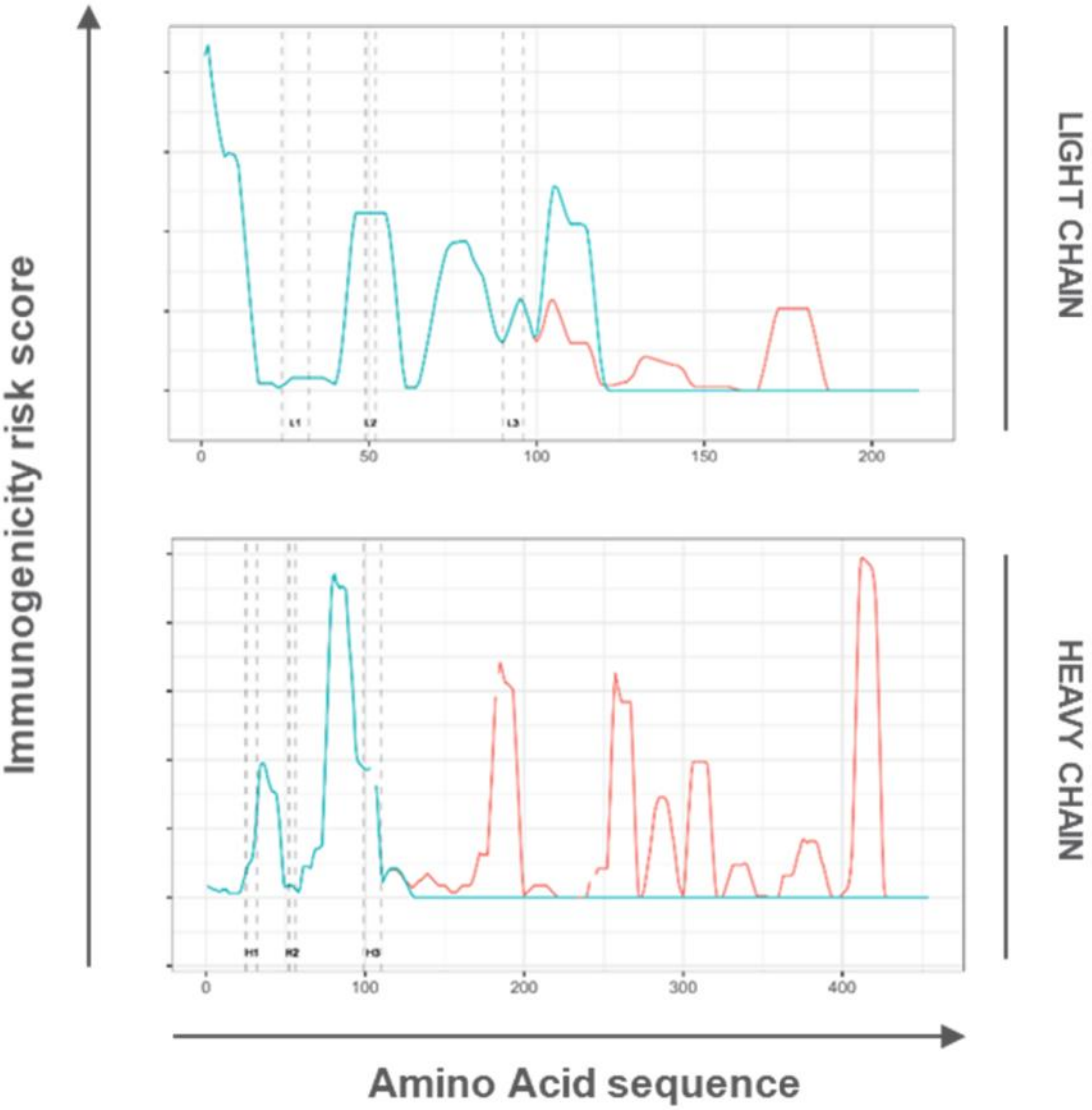

Figure S1) Predicted immunogenicity profile of NNV003. Overlapping self-adjusted predicted immunogenicity profiles of NNV003 (blue) and lilotomab (red) light (top panel) and heavy (bottom panel) chains generated using sequence position-specific immunogenicity risk score obtained from an in-silico MHC class II-peptide binding prediction analysis for the world population, performed using the algorithm NetMHCIIpan version 3.1. Grey dotted lines highlight the position of the complementary-determining regions.

Binding of ^{177}Lu-NNV003 to CD37 positive cell lines

The equilibrium dissociation constant, K_d, and the mean number of binding sites, B_{max}, were measured for ^{177}Lu-NNV003 in MEC-2, REC-1 and DOHH-2 using Scatchard analysis [6]. 10 x 10^6 cells/ml in 0.2 ml cold PBS + 0.5% BSA were incubated with ^{177}Lu-NNV003, at concentrations between 20 ng/ml and 25 µg/ml, for one hour at 4°C while shaking. Three to four replicates were made where one or two of them were blocked with 20 µg unlabelled NNV003 prior the addition of ^{177}Lu-NNV003. Cells were washed three times in cold PBS + 0.5% BSA. Activity of cells was measured before and after washing using a calibrated gamma counter (Wizard 3470, PerkinElmer, USA). The experiment was performed on 2–3 separate occasions. The results are presented in Table S1.

Table S1) Affinity (K_d) of ^{177}Lu-NNV003 and the mean number of CD37 antigens per cell (B_{max})

Cell line	B_{max} (antigens/cell)	K_d (nM)
REC-1	60 000 ± 7 200[a]	0.69 ± 0.07
MEC-2	80 300 ± 28 000[b]	1.28 ± 0.33
DOHH-2	104 000 ± 24 000[b]	1.01 ± 0.15

[a]average of two individual experiments ± SD, [b]average of three individual experiments ± SD

Internalisation of ^{177}Lu-NNV003

MEC-2, REC-1 and DOHH-2 cells were seeded in 96-well plates in their respective medium at 1 x 10^6 cells/mL, using 150 µl per well and three replicates per cell line. ^{177}Lu-NNV003 was added at a final concentration of 1 µg/ml. The plates were incubated at 5% CO_2 and 37°C for 10 min to 18 hours. After incubation, cells were washed twice, first with ice cold medium, then with PBS containing 1% FBS. The following treatment with ice cold glycine/NaCl buffer pH 2.6 for 2 min (performed twice) separated the surface bound antibody from the internalised antibody. Activity of cell pellet and acid wash supernatant was measured by gamma counter (Wizard^2 2470, PerkinElmer, USA). The results are presented in Figure S2.

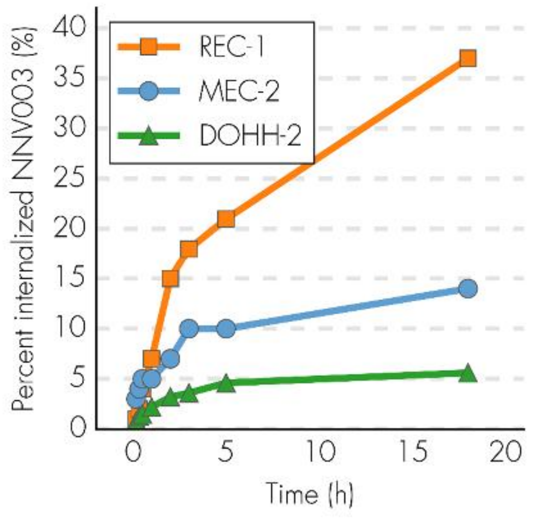

Fig S2) Percent internalization of ^{177}Lu-NNV003 in MEC-2, REC-1 and DOHH-2 cells (n=1)

Additional ^{177}Lu-NNV003 therapy studies

Female RAG-1 mice (NOD.Cg-*Rag1^{tm1Mom}Il2rg^{tm1Wjl}*/SzJ; The Jackson Laboratory, USA) were injected intravenously (i.v.) with 2.5 x 10^6 MEC-2 cells two days before treatment with ^{177}Lu-NNV003 (200 MBq/kg), unlabelled NNV003 (0.33 mg/kg), 0.9% NaCl, or unlabelled (0.33 mg/kg) or ^{177}Lu labelled (200 MBq/kg) unspecific isotype control (cetuximab) (n=8 mice in each group). Two groups were given two doses of either ^{177}Lu labelled (2 x 200 MBq/kg) or unlabelled NNV003 (2 x 0.33 mg/kg) with two weeks between the treatments. Specific activity of the RICs were 570 MBq/mg (^{177}Lu-NNV003, first injection), 625 MBq/mg (^{177}Lu-NNV003, second injection) and 480 MBq/mg (^{177}Lu-IgG1). At time of injection the mice were 8-9 weeks old and the average weight was 22 g. The day prior to injection of RIC, 200 µg murine IgG2a-κ (M7769-5MG, Sigma-Aldrich, USA) was administered intraperitoneally (i.p.) to inhibit the binding of ^{177}Lu-NNV003 to murine Fc receptors.

Female RAG-2 mice (129S6/SvEvTac-*Rag2^{tm1Fwa}*, Taconic, USA) were injected i.v. with 10 x 10^6 DOHH-2 cells three days before treatment with 200 MBq/kg (n=9), 300 MBq/kg (n=10) or 400 MBq/kg (n=10) ^{177}Lu-NNV003, 2 mg/kg (n=9) or 30 mg/kg (n=9) unlabelled NNV003, 0.9% NaCl (n=10), or 2 mg/kg unlabelled (n=9) or 300 MBq/kg ^{177}Lu labelled (n=9) unspecific isotype control (cetuximab). Specific activity of the RICs were 175 MBq/mg (^{177}Lu-NNV003) and 200 MBq/mg (^{177}Lu-IgG1). At time of injection the mice were 9-13 weeks old and the average weight was 20 g. Histopathology was performed in all mice to evaluate DOHH-2 tumour infiltration. The day prior to injection of RIC, 200 µg murine IgG2a-κ (M7769-5MG, Sigma-Aldrich, USA) was administered i.p.

In all studies, the body weights and clinical symptoms were monitored. Animals were euthanized when a humane end-point was reached: hind leg paralysis, weight loss >15% (20% of normal body weight in DOHH-2 study), palpable tumour >15 mm (two ovarian tumours = 10–12 mm or a single ovarian tumour = 20 mm in DOHH-2 study) or any signs of severe sickness or discomfort. GraphPad Prism 7.00 (GraphPad Software, La Jolla California, USA) was used to create Kaplan-Meier survival curves and to perform comparisons of the groups using Log-rank test and Holm-Sidak method with $\alpha = 0.05$.

The results from the two therapy studies are presented in Figure S3. In the MEC-2 i.v. model the median survival time of the control groups, NaCl, 0.33 mg/kg IgG1, 200MBq/kg ^{177}Lu-IgG1 and the NNV003 group was between 19 and 21 days. In the two ^{177}Lu-NNV003 groups the median survival time was extended to 32 days for the single injection group and 29 days for the repeated injection group. The survival in the ^{177}Lu-NNV003 groups was significantly better than the NNV003 groups and the ^{177}Lu-IgG1 group ($p < 0.025$), however they were not significantly better than the NaCl group ($p > 0.05$). In the DOHH-2 study, the median survival time of the control groups were 46, 47 and 49 days for the unspecific IgG1, ^{177}Lu-IgG1 and NaCl group, respectively. Survival of mice treated with NNV003 or ^{177}Lu-NNV003 at the end of the study, 219 days after cell injection, were 89-100 % (Figure S3b). Histopathology of organs of the RAG-2 mice revealed tumour infiltration in 100% of the control groups, 1/9 mouse in both NNV003 groups, 2/9 mice in the 200 MBq/kg ^{177}Lu-NNV003 group, none in the 300 MBq/kg ^{177}Lu-NNV003 group and 1/10 mouse in the 400 MBq/kg ^{177}Lu-NNV003. Tumour infiltration was generally observed in a high percentage of organs, mainly related to ovaries, bone G/I tract, lymph nodes, spleen and perirenal adipose tissues. In ^{177}Lu-NNV003 or NNV003 treated mice the number of infiltrated organs was lower than the control groups.

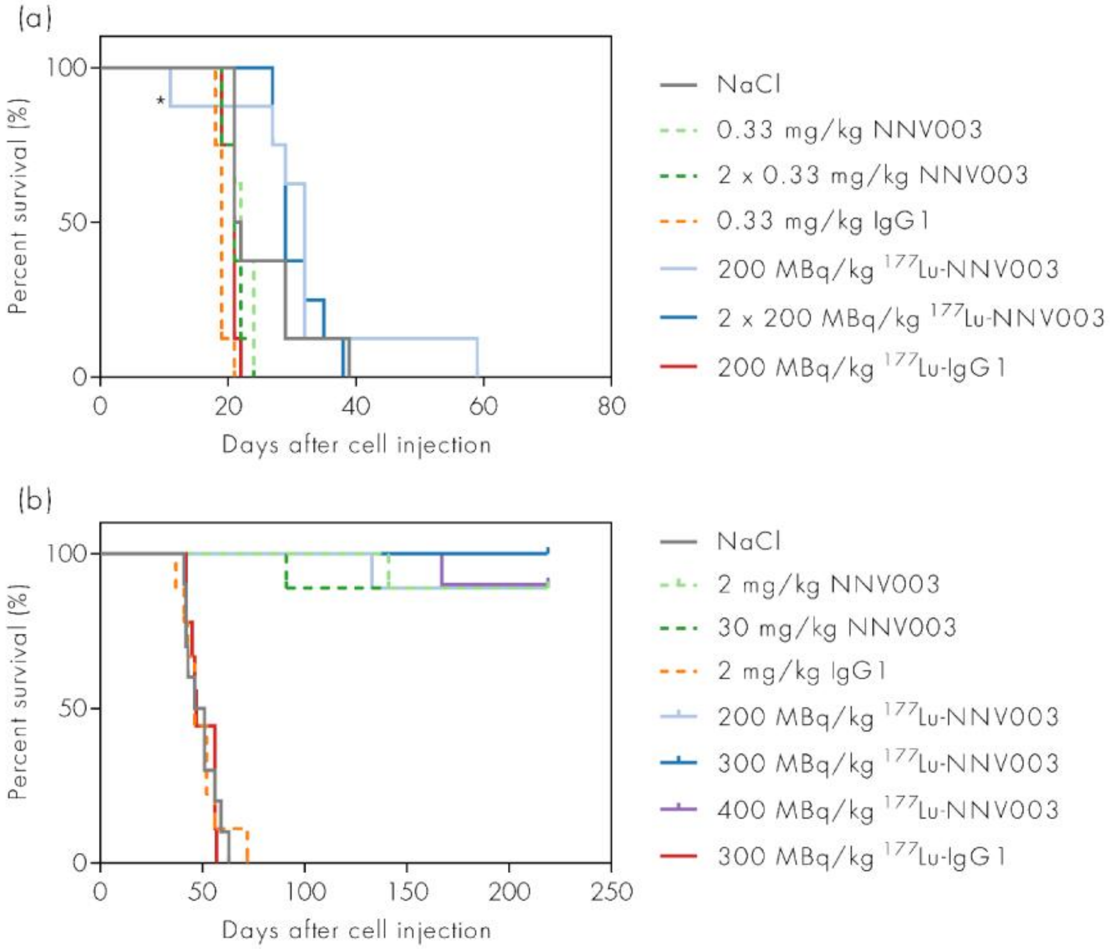

Fig S3) Survival analysis of two studies with ^{177}Lu-NNV003. (a) RAG-1 mice (n=8 per group) were injected i.v. with MEC-2 cells intravenously and (b) CB17 SCID mice (n=9 or 10) were injected intravenously with DOHH-2 cells at day 0. Therapy injections were given at day 2 (MEC-2 study) or day 3 (DOHH-2 study) and some groups (marked 2 x) received a second injection at day 9. One death in (a), marked with an asterisk, was probably caused by radiation toxicity as no macroscopic tumours were found during autopsy

References

1. Lindmo T, Boven E, Cuttitta F, Fedorko J, Bunn PA, Jr. Determination of the immunoreactive fraction of radiolabeled monoclonal antibodies by linear extrapolation to binding at infinite antigen excess. J Immunol Methods. 1984;72(1):77-89.

2. National Nuclear Data center, ENSDF Decay Data in the MIRD Format for ^{177}Lu. 2003.

3. Miller WH, Hartmann-Siantar C, Fisher D, Descalle MA, Daly T, Lehmann J et al. Evaluation of beta-absorbed fractions in a mouse model for 90Y, 188Re, 166Ho, 149Pm, 64Cu, and 177Lu radionuclides. Cancer Biother Radiopharm. 2005;20(4):436-49.

4. Andreatta M, Karosiene E, Rasmussen M, Stryhn A, Buus S, Nielsen M. Accurate pan-specific prediction of peptide-MHC class II binding affinity with improved binding core identification. Immunogenetics. 2015;67(11):641-50.

5. Hamze M, Meunier S, Karle A, Gdoura A, Goudet A, Szely N et al. Characterization of CD4 T Cell Epitopes of Infliximab and Rituximab Identified from Healthy Donors. Front Immunol. 2017;8:500.

6. Dong C, Liu Z, Wang F. Radioligand saturation binding for quantitative analysis of ligand-receptor interactions. Biophys Rep. 2015;1:148-55.
